# High−Accuracy Film−Integrated Optical Sensor for Real−Time Intraocular Pressure Monitoring

**DOI:** 10.3390/mi14020353

**Published:** 2023-01-31

**Authors:** Xiaobin Xu, Zixuan Liu, Liqiang Wang, Yifei Huang, He Yang

**Affiliations:** 1 School of Instrumentation and Optoelectronic Engineering, Beihang University, Beijing 100191, China; 2 Department of Ophthalmology, Chinese PLA General Hospital, Medical School of Chinese PLA, Beijing 100853, China

**Keywords:** glaucoma, intraocular pressure (IOP), implantable IOP sensor, interferometry

## Abstract

Intraocular pressure (IOP) is a key indicator to evaluate the risk and status of glaucoma, which is one of the main causes of irreversible blindness. However, the IOP value is susceptible to circadian changes and is difficult to be measured real−time. In this paper, we designed a thin−film integrated optical IOP sensor based on the interferometry principle, which could read out the IOP value by interference patterns and monitor the value changes real−time at the same time. The theoretical and experimental results indicated that our sensor exhibited a sensitivity of 0.19 μm/mmHg and an average accuracy of 0.84 mmHg over the pressure range of 0–45 mmHg, which is comparable with the other reported optical systems but with the advantage of easier fabrication process and low−cost. Our sensor device implies great potential in the application of human physiological index measurement and other chip−integrated medical sensing instruments.

## 1. Introduction

Glaucoma is one of the main causes of irreversible blindness [1]. It is estimated that about 111.8 million people worldwide will suffer from glaucoma by 2040 [2]. In addition to the examination of the optic nerve head and retinal nerve fiber layer changes, intraocular pressure (IOP) is another important indicator in the diagnosis of glaucoma with higher values compared with the normal data [3,4]. Therefore, it is significant to study precise clinical methods of IOP measurement for the treatment of glaucoma. Until now, Goldman applanation tonometry is one of the widely used methods for IOP measurement by the method of pressing the central corneal to a given area [5]. However, due to the different central corneal thicknesses, this method is susceptible to causing errors [6]. In addition, studies showed that the majority (67.2%) of the peak 24−h IOP values in glaucoma patients occurred at night; only 32.8% of patients experienced peak IOP during daytime [7]. Therefore, the variation of IOP peak values is easy to mislead the doctors’ diagnosis. In order to improve glaucoma care, it is important and meaningful to develop reliable IOP measurement and monitoring devices.

For the past few years, various methods based on different principles have been studied to realize real−time and reliable IOP monitoring. According to the method of pressure−sensing, IOP sensors could be classified into wearable and implantable IOP sensors [8]. The wearable IOP sensors were integrated into contact lenses to detect the pressure based on the changes of corneal curvature [8]. However, the accuracy of this measurement method was severely limited for the corneal biomechanical properties and thickness variations in the central corneal [9]. We could compare the sensitivity of implantable IOP sensors and wearable IOP sensors in the same principle, as shown in Table 1. Obviously, the average sensitivity of implantable IOP sensors is higher than that of wearable IOP sensors.

In comparison, implantable IOP monitoring devices could obtain more accurate and reliable measurement results for the direct measurement methods as the IOP sensors were implanted into eyeball structures by surgery. When the IOP varied, the flexible material was deformed, directly changing the signal standing for the intensity fluctuation of IOP. Furthermore, various measurement schemes based on electrical, microfluidic, and optical technologies have been studied. For example, Po−Jui Chen et al. reported an IOP sensor based on LCR circuit [20] with the results of 7000 ppm/mmHg sensitivity and 1 mmHg resolution [8]. Amit Todani et al. proposed an active approach based on capacitance variations [21], which gave a measurement accuracy of 0.81 mmHg. In this structure, the IOP variations changed following the value of the capacitor, which would be detected by the external reading device. Recently, some new approaches using microfluidic technology emerged with the advantage of simple structure and low cost. For example, Araci et al. reported an implantable IOP sensor integrated into an intraocular lens for IOP measurement by testing the changes in a gas−liquid interface, giving the results of an average error of ±0.5 mm Hg [22]. Though the microfluidic technology was simple and low−cost, it was still difficult to read out the pressure optically through a hazy cornea.

Recent advances in continuous IOP monitoring were optical methods, such as spectral reflectance [23], optical grating technology [24], and membrane interferometry [25] for the advantages of optical sensors in IOP measurement. Jeong Oen Lee et al. proposed an IOP sensor with an optical cavity [23] giving the higher accuracy of 0.29 mm Hg, but it required gold−nanodot arrays to strengthen the optical resonance, leading to more complicated and higher−cost fabrication. Jayer Fernandes et al. reported a new approach using optical grating technology with a pressure range of 0–50 mmHg, but the measurement accuracy is not high enough. To reduce the fabrication complexity and improve the measurement accuracy, Alex Phan et al. proposed another IOP device based on the interferometry principle showing the advantages of easier fabrication, lower cost but higher accuracy, which opened new avenues for reliable IOP monitoring [25]. However, only few works were reported demonstrating the integrated optical sensor device using this method, which still needs to be further explored.

In this work, we experimentally demonstrated an IOP monitoring system based on the optical interferometry principle by integrating the flexible polydimethylsiloxane (PDMS) membranes. Compared with the work in [25], we designed a novel wedge−cavity structure to produce vertical interference stripes, showing the relationship between the pressure and the moving distance of center fringe visually. What is more, we innovatively selected PDMS as the material of pressure−sensitive membrane. Our results showed that the IOP sensor gave an accuracy as high as 0.84 mmHg over the range of 0–45 mmHg. Simultaneously, our IOP sensor could examine the IOP values by monitoring the interference fringes distance and shape variations on the screen easily and in real−time, which provided high convenience and accuracy in the diagnosis of glaucoma. Thus, our IOP sensor shows great opportunity in the application of human physiological index measurement, optical medical instruments, water pressure−based sensing measurement, and other micro− and nano−medical integrated devices.

## 2. Principle and Design

### 2.1. System Operating Principle

**Figure 1:**
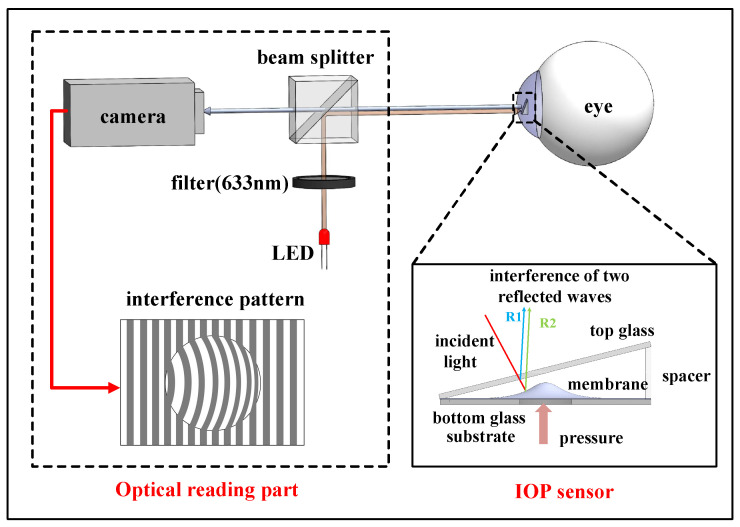
Optical intraocular pressure measurement system with implantable sensor and optical reading part.

As shown in Figure 1, the IOP measurement system consists of an implantable sensor and an optical reading part. The optical reading part includes a camera, a filter (CWL = 632.8 ± 0.2 nm), a beam splitter, and a LED (λ = 635 nm, P_power_ = 4 mW). In order to generate high−quality interference patterns in our sensor, the monochromatic light should have a long temporal coherence length. After comparison, the monochromatic light at 632.8 nm wavelength with 330 μm temporal coherence length was selected. The implantable IOP sensor is a hermetically hollow wedge cavity that includes top glass, spacer, and bottom glass substrate with a drilled hole in the center covered by polymer membrane, as demonstrated in the inset of Figure 1.

**Figure 2:**
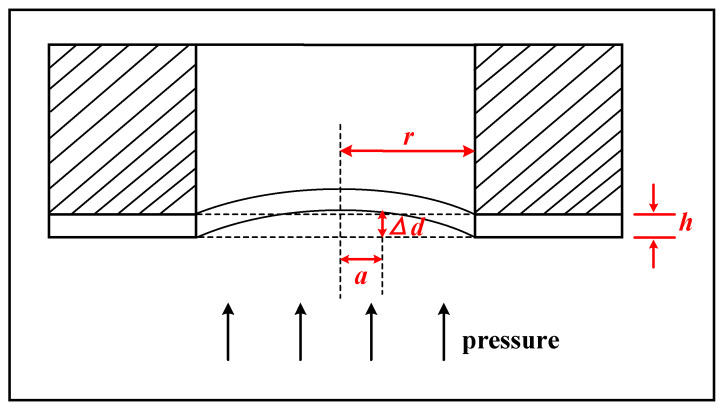
Schematic of membrane deformation under pressure.

The working principle of our IOP sensor is described as the following: the monochromatic light at the wavelength of 633 nm was divided into two beams. One beam was reflected at the undersurface of the top glass and the other was reflected at the surface of the membrane. Then, the two beams interfered at the undersurface of the top glass, producing fringes depending on the gap distance and the phase difference. Figure 2 shows the detailed interference working scheme. The function between phase difference Δ*φ(x, y)* and gap distance *d(x, y)* between the top glass and membrane could be expressed as [25]:

(1) $$\Delta \varphi (x,y)=\frac{4\pi }{\lambda }d(x,y)+\pi$$

where *λ* is the wavelength of the incident light, π is the phase shift occurring at the top surface of the diaphragm, *d(x, y) = h −* Δ*d* means the gap distance, *h* is the spacer thickness and Δ*d* is the deflection of the diaphragm. The polymer membrane is flexible, serving as a pressure−sensitive element by detecting the value of Δ*d*. With the increase of the pressure, the deflection of the membrane increases, corresponding to the increase of Δ*d* shown in Figure 2.

According to the principle of elasticity, if the maximum deflection under pressure is not more than 30% of the membrane thickness and, at the same time, the membrane thickness is not larger than 20% of the diameter, the deflection Δ*d* of a diaphragm with fixed edges under pressure (*P*) at any radial distance (*a*) could be expressed as [26]:

(2) $$\Delta d=\frac{3}{16}\frac{(1-\mu^{2}){\left(r^{2}-a^{2}\right)}^{2}}{Eh^{3}}P$$

where Δ*d, μ, r, a, E, h*, and *P* are the deflection, Poisson’s ratio of the diaphragm, radius of the diaphragm, radial distance, modulus of elasticity, thickness, and pressure, respectively. Based on Equations (1) and (2), the precise pressure applied to the membrane could be calculated.

### 2.2. Design of the IOP Sensor

**Figure 3:**
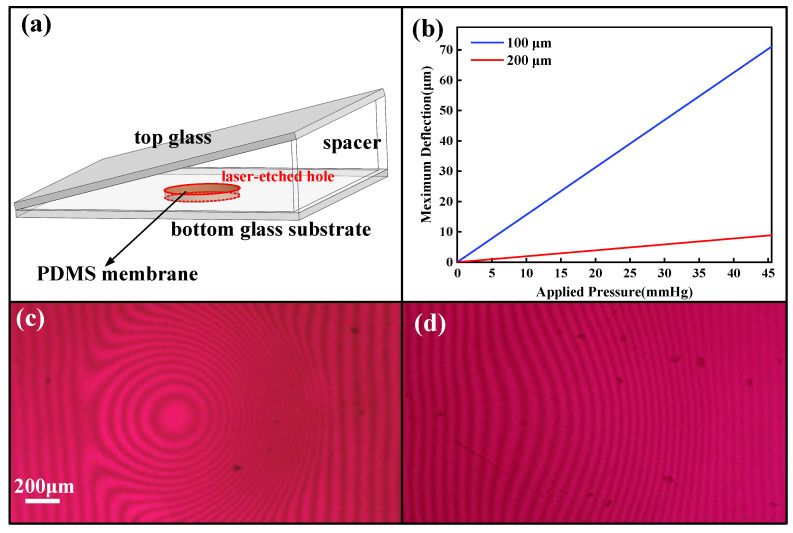
(**a**) The structure of IOP sensor. (**b**) Simulation of 100 μm− and 200 μm−thick membranes deflection under pressure. (**c**) Interference pattern of 100 μm−thick membrane under the pressure of 22.5 mmHg. (**d**) Interference pattern of 200 μm−thick membrane under the same pressure.

The detailed structure of IOP sensor is shown in Figure 3a. In order to guarantee the high sensitivity, accuracy, and safety of IOP sensor, the membrane material should be flexible and biocompatible. PDMS is a kind of biocompatible material that is widely used in biomedical devices and its elasticity modulus of PDMS is 750 K, which is much lower than other flexible materials. What is more, PDMS membrane is also low−cost and easy−fabricated. Considering the flexibility and biocompatibility of the membrane, PDMS was selected as the membrane material to detect the pressure. Following Equation (2), as the diameter of the hole was 1 mm, the membrane thickness should not be more than 200 μm. To select the most appropriate membrane, we compared the deflection results from two membranes with different thicknesses of 100 μm and 200 μm under the same pressure, respectively. Figure 3b shows the simulated curve of the membrane deflection that the defection is more sensitive with the PDMS thickness at 100 μm compared with that of 200 μm−thick PDMS membrane. According to Equation (2), the thinner membrane had a larger deflection under the same pressure, which was consistent with our simulation results. In our experiments, by setting the pressure at 22.5 mmHg, the interference patterns resulting from the membrane thickness at 100 μm and 200 μm also showed huge differences, as shown in Figure 3c,d separately. Compared with the two figures, 100 μm−thick PDMS membrane had sharper deflection and the stripes became circles. While, the 200 μm−thick PDMS membrane had a measurable moving distance of stripes. For the convenience of our experiment, the higher deflection sensitivity from the membrane was not easy for the algorithm to distinguish the transverse displacement and results error. Thus, the PDMS membrane with the thickness of 200 μm was selected in our experiment to ensure the accuracy of the results. Considering the above all, our sensor was finally constructed with the PDMS membrane at 200 μm thickness, the spacer height at ~85 μm, the laser−drilled hole diameter at 1 mm, and the size of the holding substrate of 5 mm × 5 mm.

### 2.3. Algorithm

**Figure 4:**
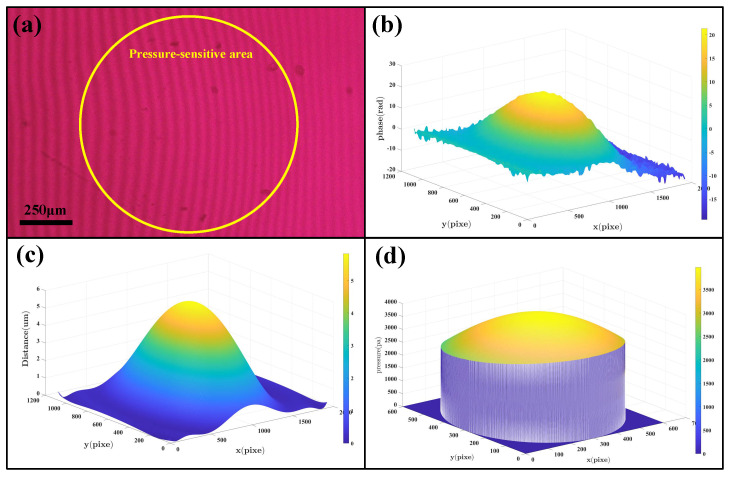
(**a**) Interference pattern at 22.5 mmHg. (**b**) Phase diagram of Interference pattern. (**c**) 3D model of the membrane. (**d**) The distribution diagram of pressure on the membrane.

To analyze the interference patterns, we developed a unique image−processing algorithm to reconstruct the 3D model of membrane deflection and calculate the pressure according to Equations (1) and (2). To test the performance of the algorithm, the interference pattern generated under the pressure of 3 Kpa (22.5 mmHg) was selected for the algorithm analysis, as shown in Figure 4a. Firstly, the interference pattern was transformed into the frequency domain by Fourier Transformation. Then, we could obtain the phase diagram from the filtered figures (Figure 4b). Using Equations (1) and (2), the 3D model of the membrane defection and the pressure distribution diagram on the membrane was constructed, as illustrated in Figure 4c,d separately. Lastly, the pressure applied could be determined with a high degree polynomial fitting. Figure 4d showed that the pressure was not uniformly distributed across the membrane and the highest pressure with the value of 2.9 Kpa was located at the center of the membrane. Since the edge of the membrane was fixed, the pressure on the edge of the membrane was nearly 1 Kpa (~7.5 mmHg) less than that at the center of the membrane.

## 3. Results and Discussion

**Figure 5:**
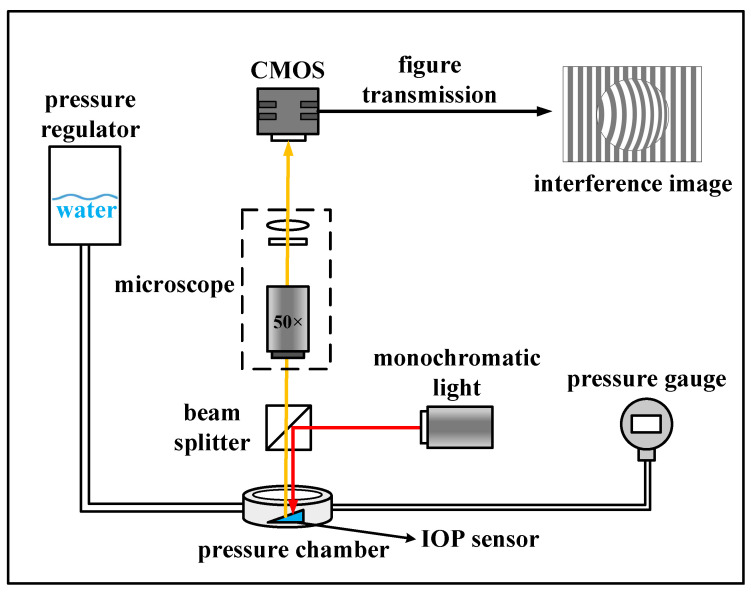
The setup of the experiments of sensor performance testing.

To test the performance of our designed sensor, we built the experimental sensing setup shown in Figure 5. The IOP sensor was fixed in a pressure chamber which was customized to mimic the environment inside the human eye. An infusion bag was connected to the pressure chamber to alter the inner pressure by adjusting the height. A standard pressure sensor was used to record the pressure changes. The camera was mounted to a microscope to capture the interference patterns of the IOP sensor. When varied pressure was applied to the sensor, the interference patterns were captured and processed by MATLAB image analysis algorithms.

**Figure 6:**
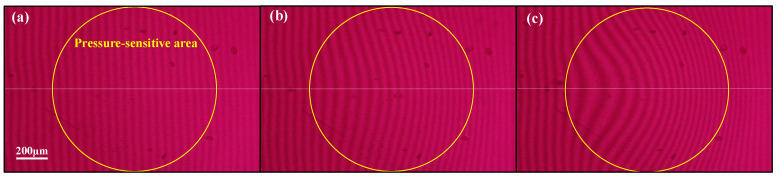
Images of interference patterns at pressure load of (**a**) 0 mmHg, (**b**) 22.5 mmHg, (**c**) 45 mmHg.

The accuracy, range, and linearity of the IOP sensor were measured inside the controlled pressure chamber filled with water. As shown in Figure 6, the fringes moved far away from the center area when the pressure inside the chamber increased from 0 to 45 mmHg at the steps of 3.75 mmHg. Figure 6a shows the interference patterns at the pressure load of 0 mmHg. When no pressure was loaded inside the pressure chamber, the interference fringe stayed vertically. As the hydrostatic pressure increased, fringes deformed sharply towards the right, which was demonstrated in Figure 6b,c.

**Figure 7:**
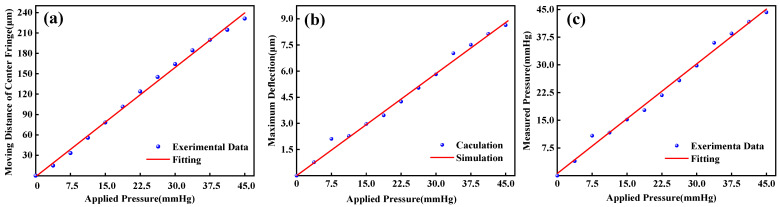
(**a**) Moving distance of center fringe as a function of applied pressure. (**b**) Maximum deflection of the sensor diaphragm and the ideal curve. (**c**) Measured pressure versus applied pressure using the IOP sensor.

We also investigated the relationship between the moving distance of the center fringe of the membrane and the pressure applied. In the experiment, we found that when the pressure was applied at the step of 3.75 mmHg, the central fringe moved at the corresponding step of ~19.27 μm toward the right. Fitting the relationship between the moving distance and the applied pressure, we could obtain a linear fitting curve with a specific value of 0.99604, as shown in Figure 7a.

At the same time, we adopted our image processing algorithm to analyze the interference patterns and calculated the maximum deflection of the PDMS membrane, which was plotted by dots in Figure 7b. The simulated curve of the 200 μm−thick membrane deflection was also plotted by the red line in Figure 7b. The simulated line was fully following the experimental results with the average error of ±0.16 μm considering the dimensional error of laser−etched hole radius and membrane thickness. Thus, the sensitivity of our sensor of 0.19 μm/mmHg could be determined. Further work will be performed to improve the sensor sensitivity by modifying the size of the sensor, such as reducing the thickness of the membrane and shrinking the radius of the laser−etched hole.

Figure 7c shows the relationship between the pressure value analyzed by the image processing algorithm and the applied pressure from the infusion bag, where the red line is fitted by a third−order polynomial curve on the experimental data. The experimental data was plotted by dots which had a linear relationship with calculated deflection data shown in Figure 7b. On the other hand, it showed that the IOP sensor had highly linear responses, and the average error from the standard pressure−gauge reading was 0.84 mmHg, which further verify the high quality or reliability of our constructed sensor. A performance comparison with previous reports is shown in Table 2.

In further studies, we would make more efforts to improve the portability of the IOP monitoring system and increase its accuracy. In this study, the factors affecting the accuracy of IOP sensors include manufacturing error and image definition. To increase the accuracy of IOP sensor, we will use MEMS technology to reduce manufacturing errors during the fabrication process of IOP sensor. In experiments, the accuracy of the image processing algorithm is susceptible to image definition. We could improve image definition by using a monochromatic light source with longer temporal coherence length and higher pixel cameras. To allow patients to realize IOP self−monitoring anywhere and at any time, the optical reading part would be integrated into an adapter that could be installed on smartphones. In the future, the IOP sensor could be integrated on smaller substrate and bonded to intraocular lenses to guarantee safety and biocompatibility.

## 4. Conclusions

In this work, we constructed an optical implantable sensor system based on the interferometry principle for continuous and real−time IOP monitoring. The sealed wedge cavity and pressure−sensitive membrane in the structure were designed to improve the detection sensitivity of IOP. As the variation of the IOP could be detected in real−time by monitoring the phase change from the interference patterns, no external power source was required. Our designed IOP monitoring system shows a sensitivity as high as 0.19 μm/mmHg and an average accuracy of 0.84 mmHg. Thus, it shows great reliability in the diagnosis and treatment of glaucoma patients as an implantable medical device, providing a warning of danger in the range of high IOP value. Our work also implied that the optical IOP monitoring systems would make more contributions in relieving the glaucoma patient’s indisposition and providing more sensitive and accurate IOP information to advance the development of the optical sensors.

## Figures and Tables

**Table 1:** Comparison of the sensitivity of inductive couple telemetry−based wearable IOP sensors and implantable IOP sensors.

Wearable IOP Sensors		Implantable IOP Sensors	
Ref.	Sensitivity	Ref.	Sensitivity
[10]	~2.2 MHz mmHg^−1^	[11]	243 kHz mmHg^−1^
[12]	23 kHz mmHg^−1^	[13]	1.14 MHz mmHg^−1^
[14]	8 kHz mmHg^−1^	[15]	119.88 kHz mmHg^−1^
[16]	57 kHz mmHg^−1^	[17]	120 kHz mmHg^−1^
[18]	35.1 kHz mmHg^−1^	[19]	156 kHz mmHg^−1^

**Table 2:** Comparison of our implantable IOP sensor with previous reports.

Ref.	Working Principle	Sensitivity	Accuracy	Merit and Demerit
[20]	Inductive	7000 ppm/mmHg	Not mentioned	Merit: high sensitivity Demerit: non−portability of the external reading device
[21]	Capacitive	Not mentioned	0.81 mmHg	Merit: long−term implantation of IOP detector in animal experimentations will not produce inflammation. Demerit: the size is too large to be implanted in humans’ eye
[22]	Microfluidic	106 μm/mm Hg	0.5 mmHg	Merit: simple and low−cost Demerit: it is difficult to read out the pressure optically through a hazy cornea
[23]	Spectral reflectance	Not mentioned	0.29 mmHg	Merit: high accuracy Demerit: more complicated and higher−cost fabrication
[25]	interferometry	31 nm/mmHg	0.3 mmHg	Merit: high accuracy and sensitivity Demerit: more complicated fabrication
This work	interferometry	0.19 μm/mmHg	0.84 mmHg	Merit: easy fabrication process and low cost

## Data Availability

Data presented in this study are available on request from the corresponding author.
